# Pan-cancer analysis of promoter activity quantitative trait loci

**DOI:** 10.1093/narcan/zcad053

**Published:** 2023-11-14

**Authors:** Ran Li, Dongyi Wan, Junnan Liang, Huifang Liang, Haohao Huang, Ganxun Li

**Affiliations:** Department of Neurosurgery, Tongji Hospital, Tongji Medical College, Huazhong University of Science and Technology, Wuhan, Hubei, 430000, China; Department of Nuclear Medicine, Tongji Hospital, Tongji Medical College, Huazhong University of Science and Technology, Wuhan, Hubei, 430000, China; Hepatic Surgery Center and Hubei Key Laboratory of Hepato-Biliary-Pancreatic Diseases, Tongji Hospital, Tongji Medical College, Huazhong University of Science and Technology, Wuhan, Hubei, 430000, China; Hepatic Surgery Center and Hubei Key Laboratory of Hepato-Biliary-Pancreatic Diseases, Tongji Hospital, Tongji Medical College, Huazhong University of Science and Technology, Wuhan, Hubei, 430000, China; Department of Neurosurgery, General Hospital of Central Theatre Command of People’s Liberation Army, Wuhan, Hubei, 430000, China; Hepatic Surgery Center and Hubei Key Laboratory of Hepato-Biliary-Pancreatic Diseases, Tongji Hospital, Tongji Medical College, Huazhong University of Science and Technology, Wuhan, Hubei, 430000, China

## Abstract

Altered promoter activity has been generally observed in diverse biological processes, including tumorigenesis. Accumulating evidence suggests that employing a quantitative trait locus mapping approach is effective in comprehending the genetic basis of promoter activity. By utilizing genotype data from The Cancer Genome Atlas and calculating corresponding promoter activity values using *proActiv*, we systematically evaluated the impact of genetic variants on promoter activity and identified >1.0 million promoter activity quantitative trait loci (paQTLs) as both *cis-* and *trans*-acting. Additionally, leveraging data from the genome-wide association study (GWAS) catalog, we discovered >1.3 million paQTLs that overlap with known GWAS linkage disequilibrium regions. Remarkably, ∼9324 paQTLs exhibited significant associations with patient prognosis. Moreover, investigating the impact of promoter activity on >1000 imputed antitumor therapy responses among pan-cancer patients revealed >43 000 million significant associations. Furthermore, ∼25 000 significant associations were identified between promoter activity and immune cell abundance. Finally, a user-friendly data portal, Pancan-paQTL (https://www.hbpding.com/PancanPaQTL/), was constructed for users to browse, search and download data of interest. Pancan-paQTL serves as a comprehensive multidimensional database, enabling functional and clinical investigations into genetic variants associated with promoter activity, drug responses and immune infiltration across multiple cancer types.

## Graphical Abstract

**Graphical Abstract:**
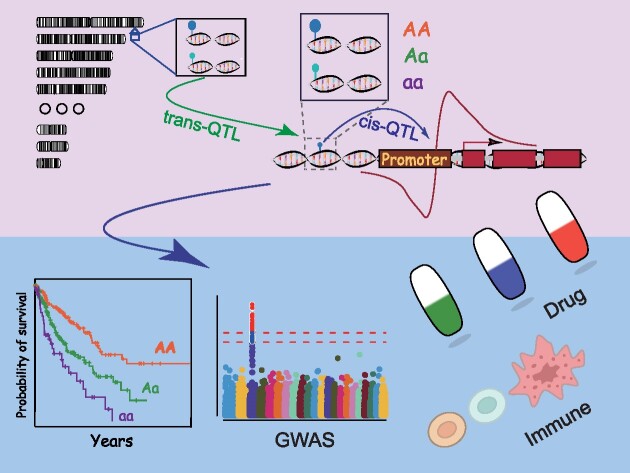

## Introduction

Located upstream of transcription start sites, promoter elements play a crucial role in regulating the initiation and activity of transcription. They integrate signals from proximal epigenetic modifications and distal regulatory elements. In humans, the transcriptional initiation of most protein-coding genes is controlled by multiple promoters, leading to the production of distinct gene isoforms (1,2). Unlike alternative splicing, which modulates gene isoform expression post-transcriptionally, the usage of alternative transcription start sites allows for pre-transcriptional control of gene isoform expression (3). Consequently, promoters not only regulate the activity and level of gene expression, but also determine which gene isoforms are expressed. Previous studies have demonstrated that alternative promoters often function in a tissue- or cell-specific manner, and dysregulation of promoters has been identified in various human diseases (4,5). Somatic mutations, genomic rearrangements and alternations in regulatory or epigenetic landscapes have been shown to impact the promoters of several oncogenes in tumors, indicating that promoters play a role in the malignant transformation of cells (6).

Single nucleotide polymorphisms (SNPs), the most common type of genetic variant, contribute to the diversity in human disease susceptibility (7). Genome-wide association studies (GWAS) have identified thousands of SNPs that are associated with complex human traits and diseases (8). However, the underlying biological mechanisms behind these associations are not yet fully understood, and most studies currently focus on statistical significance (9). Quantitative trait locus (QTL) analysis is an approach used to investigate the impact of genetic variants on intermediate molecular phenotypes. It has proven to be a powerful method for deciphering the role of SNPs and prioritizing genetic variants within GWAS loci (10). Recent studies have highlighted the significance of promoter activity QTLs (paQTLs) in elucidating the relationship between genetic variation and promoter activity (11–13). The *cis-*regulatory mechanism of promoter activity has been studied in both cancer and normal human tissues. For example, an intergenic variant, rs8028374 (A/G), was identified as a paQTL associated with the outermost promoters of the *TTC23* gene in Epstein–Barr virus-transformed lymphoblastoid cell lines (13). Epigenomic promoter activity has also been shown to predict the response to immune checkpoint inhibition in metastatic gastric cancer (14). In addition, alternative promoter may also impact the response to anticancer drugs (15). In spite of accumulating evidence on the critical impacts of promoters in carcinogenesis and clinical utility, the landscape of correlation between promoter activity, genetic variants, response of anticancer drugs in patients with cancer and immune infiltration remains largely unexplored. Recently, Demircioğlu *et al.* developed a novel algorithm, named *proActiv*, to estimate the promoter activity in >10 000 RNA-seq samples across 33 cancer types in The Cancer Genome Atlas (TCGA) (16). This makes it feasible to extend the analysis of promoter activity to an additional dimension in current cancer genomic studies.

To fill this gap, we developed a computational pipeline to conduct paQTL analysis across 33 cancer types in TCGA. Our study identified paQTLs associated with patient prognosis and paQTLs within linkage disequilibrium (LD) regions of GWAS. In addition, we investigated the paQTL–drug associations and paQTL–immune cell associations across different cancers. Finally, we constructed a user-friendly database, Pancan-paQTL (https://www.hbpding.com/PancanPaQTL/), to provide convenient browsing, searching and downloading of paQTL-related data of interest for users.

## Materials and methods

### Data collection

The prognostic, genotype (level 2) and junction files (level 2) quantified by STAR of >10 000 tumor samples were downloaded from the TCGA data portal (https://portal.gdc.cancer.gov/). Promoter activity, defined as the total amount of transcription initiated at each promoter, was estimated using *proActiv* with aligned reads as input (https://github.com/GoekeLab/proActiv). We specifically employed the promoter activity associated with the primary or default promoter of genes with multiple alternative promoters. Distribution of absolute promoter activity across cancer types is displayed in Supplementary Figure S1. The data of immune cell infiltration were calculated using three different algorithms, including *TIMER*, *MCP-counter* and *ImmuCellAI* (17–19). The imputed GDSC (Genomics of Drug Sensitivity in Cancer; https://www.cancerrxgene.org/) drug response from cancer cell lines to TCGA patient samples was estimated using *oncoPredict* R package (20).

### Identification of paQTLs

In order to increase the statistic power of paQTL identification, BEAGLE (version 5.4) was used to impute genetic variants of TCGA tumor samples with the reference panel of the 1000 Genomes Project. The imputation parameters including minor allele frequency >5%, missing rate of SNPs <5% and Hardy–Weinberg equilibrium *P*-value >1 × 10^−4^ were used as the cutoff to detect high-confidence SNPs. Covariates including the top five principal components by PLINK, top 10 PEER factors by PEER software (https://github.com/PMBio/peer), patient age and gender, and tumor grade and stage regarded as confounder factors were also estimated to decrease potential impacts from population structure, batch effect and clinical characteristics for each tumor type. The promoter activity matrix, genotype and covariates were then used to detect paQTLs based on a linear regression model by Matrix eQTL. We defined SNPs with false discovery rates (FDRs) <0.05 as paQTLs. *cis*-paQTLs were ascertained when the SNP was located within 1 Mb from the specific promoter, and *trans*-paQTLs were identified when the SNP was beyond that location. On the basis of tag SNPs derived from the GWAS Catalog website (http://www.ebi.ac.uk/gwas/) and the LD calculated by PLINK in the 1000 Genomes Phase 3 European population, *cis-* or *trans*-paQTLs that were in LD relation (*r*^2^ > 0.5) with tag SNPs were defined as GWAS-paQTLs. Both *cis-* and *trans*-paQTLs were retrieved to investigate their potentially prognostic value by a log-rank test, with FDR < 0.05 regarded as statistical significance (Supplementary Figure S2).

### Identification of paQTL–drug response associations

The R package *oncoPredict* was used to predict *in vivo* drug responses in cancer patients on the basis of linear ridge regression analysis, which fits the gene expression matrix of samples into the half-maximal inhibitory concentration of antitumor drugs originated from GDSC (https://www.cancerrxgene.org/). Then, the associations between promoter activity of each *cis-* or *trans*-paQTL and imputed drug response were investigated by Spearman’s correlation analysis with FDR < 0.05 regarded as statistical significance (Supplementary Figure S2).

### Identification of paQTL–immune cell associations

The associations between promoter activity of each *cis-* or *trans*-paQTL and immune cell abundance calculated by *TIMER* (17), *MCP-counter* (19) and *ImmuCellAI* (18) were evaluated by Spearman’s correlation analysis with FDR < 0.05 regarded as statistical significance in each cancer type (Supplementary Figure S2).

### Database construction

The Pancan-paQTL database was constructed using JavaScript and HTML5 for the front end, and Rscript 4.2.1 and PHP 7.4 for the backend. All data resources in the Pancan-paQTL database were stored as *.gz or *.RData files. Data retrieval or processing was performed using Rscript on our server. The httpd service was running on Apache 2.4. The database can be accessed freely at http://www.hbpding.com/PancanPaQTL.

### Data availability statement

All data are available through our data portal (http://www.hbpding.com/PancanPaQTL).

## Results

### Data summary

**Figure 1.**
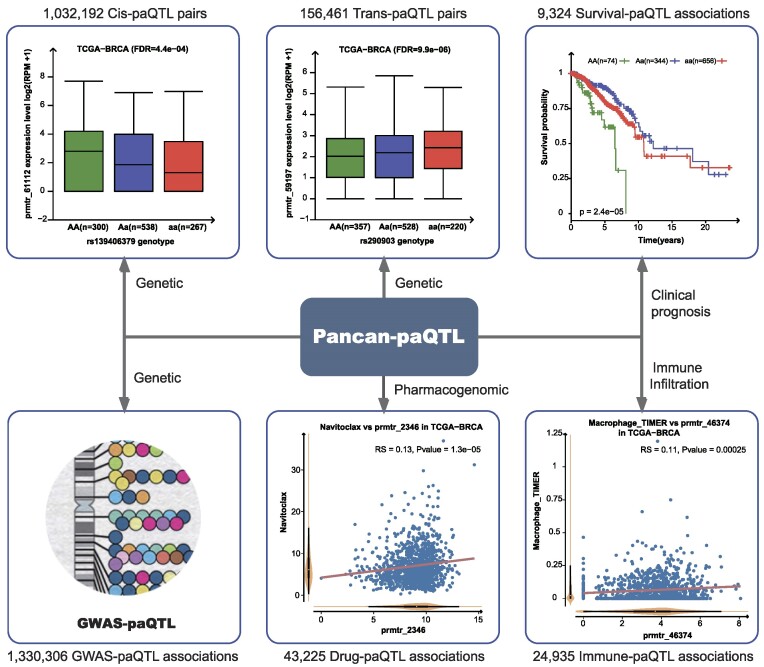
The sketch map summarizing data resources in Pancan-paQTL, with the genetic, pharmacogenomic and immune landscapes of promoter activity in a large number of patient samples, which may contribute to cancer clinical utility, including prognosis, target therapy and immunotherapy. Pancan-paQTL includes 1 032 192 *cis-*paQTL pairs, 156 461 *trans*-paQTL pairs, 9324 survival-associated paQTL pairs and 1 330 306 GWAS-associated paQTL pairs. Pancan-paQTL also covered 43 225 drug–paQTL associations and 24 935 immune cell–paQTL associations.

Distance between QTLs and promoters <1 Mb is regarded as *cis-*regulation and beyond this point regarded as *trans*-regulation, since within 1 Mb genomic region they can randomly encounter each other in the nucleus. *cis*-Acting QTLs generally modulated nearby targets by impacting chromatin structure or accessibility. *trans*-Acting QTLs have demonstrated to involve in the complicated biological processes, by altering chromatin 3D interaction, changing the expression level of nearby genes and modulating regulatory circuits dominating subsets of genes. In our analysis, 1 032 192 *cis*-paQTL pairs, 156 461 *trans*-paQTL pairs and 9324 survival-associated paQTLs were identified (Figure 1 and Supplementary Table S1). Recent studies have demonstrated that alternative promoter is an important regulator in tumor-related gene (e.g. *SEPT9* and *CTNNB1*) and tumor signaling pathways (e.g. *NF-κB* and *STING*), indicating that paQTLs might impact patient prognosis by regulating promoter activity of cancer-related genes or cancer-related pathways. Number of paQTL pairs is significantly associated with the number of patient samples across cancers (*cis-*, RS = 0.78, *P* = 8 × 10^–7^, Supplementary Figure S3A; *trans*-, RS = 0.47, *P* = 0.0063, Supplementary Figure S3B). These findings suggest that a larger cohort of patients can improve the detection of paQTLs. Importantly, these significant correlations remain even after excluding breast invasive carcinoma samples (Supplementary Figure S4A and B). Most *cis*-paQTL pairs were detected in thyroid carcinoma (*n* = 39 504), most *trans*-paQTL pairs were identified in adrenocortical carcinoma (ACC; *n* = 6264) and most survival-associated paQTL pairs were detected in cholangiocarcinoma (*n* = 981) (Supplementary Table S1). In addition, we also linked paQTL results to the NHGRI GWAS Catalog profile and identified >1.3 million paQTLs that overlapped with GWAS LD regions (*r*^2^ ≥ 0.5) of one or multiple human traits, of which the highest is 187 257 in pancreatic adenocarcinoma and the lowest is 22 441 in breast invasive carcinoma (Supplementary Table S1).

In anticancer drug response analysis, we calculated the correlations between promoter activity and imputed drug response across 33 TCGA cancer types, which offer much more direct and significant effect of promoter activity in anticancer therapy than *in vitro* cell models. In total, 43 225 correlations between promoter activity and impute drug responses were identified in our analysis (Figure 1 and Supplementary Table S1). The number of paQTL–drug correlations ranged from 376 in thyroid carcinoma to 3188 in skin cutaneous melanoma (Supplementary Table S1).

Current studies have demonstrated the important function of promoter activity in modulating the infiltration of immune cells and the efficiency of checkpoints. The correlations between promoter activity and the infiltration of immune cells were also investigated in our analysis, which provides potential paQTL-based biomarkers for tumor immunotherapy. We identified 24 935 correlations between promoter activity and the infiltration of immune cells, including 2989 correlations from *TIMER*, 7891 associations from *ImmuCellAI* and 14055 associations based on *MCP-counter* (Figure 1 and Supplementary Table S1). The number of paQTL–immune cell correlations ranged from 73 in glioblastoma multiforme to 2507 in colon adenocarcinoma (Supplementary Table S1). All above identified pairs were systematically organized in the Pancan-paQTL database. All related results for each cancer type are available for downloading at http://www.hbpding.com/PancanPaQTL/download.

### Database access

**Figure 2.**
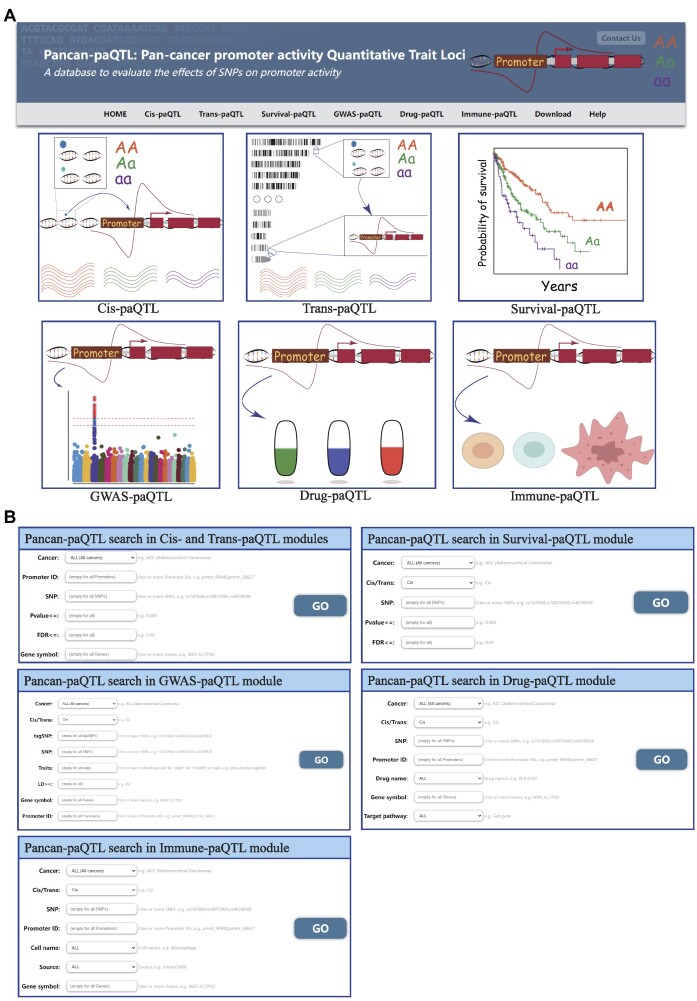
Content and interface of Pancan-paQTL. (**A**) Home page and six main modules of Pancan-paQTL. Modules are placed on both header and module logo on home page, and clicking either header or logo could step in the related module. (**B**) Search boxes in each module.

Pancan-paQTL has six modules: Cis-paQTL, Trans-paQTL, Survival-paQTL, GWAS-paQTL, Drug-paQTL and Immune-paQTL (Figure 2A). On the home page, by clicking on the corresponding button in the browser bar, users can enter the ‘Cis-paQTL/Trans-paQTL/Survival-paQTL/GWAS-paQTL/Drug-paQTL/Immune-paQTL’ pages (Figure 2A). By querying the Cis-/Trans-paQTL page, users could acquire a table including the information of the ID, genomic position and alleles of specific SNPs, the ID, gene symbol and genomic position of specific promoters, and beta value (effect size of SNP on promoter activity), *t*-stat value, *P*-value and FDR of paQTLs (Figure 2B). For each query, a vector diagram of the boxplot was embedded to present the correlation between SNP genotypes and promoter activity. By retrieving the Survival-paQTL page, users could acquire a table including the information of the ID, genomic position and alleles of specific SNPs, log-rank test *P*-value and median prognostic time of different genotypes. For each query, a vector diagram of the Kaplan–Meier plot was offered for illustrating the correlation between SNP genotypes and prognosis (Figure 2B). On the ‘GWAS-paQTL’ page, users could get a table of paQTLs overlapping the LD regions of GWAS risk variants and the LD information between paQTLs and GWAS risk variants, including *R*^2^ and the associated traits (Figure 2B). The Drug-paQTL module has a search box, in which users can select a cancer type, regulation type of paQTL, SNP ID, promoter ID, promoter of gene symbol, drug name and drug target pathway for significant associations (Figure 2B). The Immune-paQTL module also has a search box, in which users can select a cancer type, regulation type of paQTL, SNP ID, promoter ID, promoter of gene symbol, immune cell and sources of immune cell infiltration data for significant associations (Figure 2B). For example, when the user selects ACC and inputs promoter ID prmtr_49950 in each module, Pancan-paQTL will return 14 *cis*-paQTL pairs, 33 GWAS-paQTL pairs, 14 drug–paQTL pairs, 20 immune-associated paQTL pairs and related diagrams (Supplementary Figure S5). While the user selects ACC and inputs SNP ID rs3008666 in each module, Pancan-paQTL will return one *cis*-paQTL pair, one GWAS-paQTL pair, one drug–paQTL pair, one immune-associated paQTL pair and related diagrams (Supplementary Figure S6).

The outputs from all analyses, including tables and high-resolution figures, can be easily downloaded from the Pancan-paQTL database. The ‘Help’ page provides the basic information on database, pipeline of database construction, result summary and contact. Pancan-paQTL is open to any feedback with email address provided at the top of the ‘Home page’.

## Discussion

Alternative promoters play a crucial role in regulating transcription and have been found to be more accurate predictors of patient survival than gene expression in cancer (16). Understanding the significance of paQTLs in relation to genetic variation and promoter activity has been a focus of several studies, with investigations primarily conducted in cancer cell lines and normal human tissues (11–13). Notably, epigenomic alterations in promoters have shown predictive value for the response to immune checkpoint inhibition in metastatic gastric cancer (14). In addition, promoter alterations have the potential to impact drug response (15), although the associations between drug response and promoter activity in a large number of cancer samples have not been explored. Despite the importance of understanding the genetic, immunological and pharmacological landscape of promoter activity in human cancers, there is currently a lack of data resources that provide comprehensive paQTL information, as well as immune and drug sensitivity relations of alternative promoters on a large scale.

To address these gaps, our analysis comprehensively integrated promoter activity with genetic variants, anticancer drug response and immune cell infiltration across human cancers, resulting in the identification of millions of promoter activity associations. To facilitate access to this valuable information, we developed the user-friendly Pancan-paQTL database, which allows users to query, browse and download promoter activity-correlated genetic variants, anticancer drug response and immune infiltration landscapes across multiple cancer types. The complex nature of gene modulation and the challenge of linking genetic variants to phenotypes necessitate further exploration. Within Pancan-paQTL, we identified >1 million paQTLs and evaluated their correlations with patient prognosis, offering a valuable resource for investigating human tumor genetics. This includes uncovering novel mediation mechanisms between variants and promoter activity to better understand the impact of variants on phenotypes, as well as shedding light on the role of genetic variants in promoter activity and their underlying functions in tumor prognosis and diagnosis.

Increasing evidence indicated that promoter activity, or active promoters, may affect the response to anticancer therapy. However, the associations between drug response and promoter activity in a large number of cancer samples have not been extensively investigated. Estimating drug response in cancer patients can provide a more direct and significant measure for antitumor treatment. In our analysis, we utilized the *oncoPredict* method to estimate drug response in cancer patients, leading to the identification of numerous paQTL–drug correlations between paQTLs and imputed drug response across 33 cancer types. It is important to note that the TCGA data resource is useful for characterizing initial cohorts, but it may differ from the patient population in clinical trials, which often consists of heavily pretreated individuals with distinct molecular features.

Immunotherapy has demonstrated significant clinical benefits in various cancer types, and efforts are ongoing to enhance its effectiveness by understanding the tumor immune microenvironment and the underlying mechanisms of checkpoints (21,22). Recent studies have highlighted the explanatory power of alternative promoters in addressing intratumoral heterogeneity in terms of T-cell cytolytic activity and T-cell abundance (23). Signatures based on promoter activity have shown potential for predicting the benefit from immune checkpoint inhibition in metastatic gastric cancer (14). Furthermore, promoter activity may differ across different infiltrating cell types (23). Therefore, the identification of paQTL–immune cell correlations can contribute to the identification of promoter-based biomarkers for immunotherapy.

In conclusion, Pancan-paQTL is a large-scale and multidimensional database that facilitates functional and clinical explorations of promoter activity across human cancers. We will continue to update Pancan-paQTL with the release of additional associated datasets, ensuring its relevance and usefulness for future research endeavors.

## Supplementary Material

zcad053_Supplemental_Files

## Data Availability

Pancan-paQTL code is publicly available and can be found on GitHub alongside any appropriate tutorials: https://github.com/wolfgangsk07/PancanPaQTL-code (permanent DOI: https://doi.org/10.6084/m9.figshare.24460201.v1).
