# Supplementary material for: Pan-cancer analysis of promoter activity quantitative trait loci
Source: NAR Cancer. 2023 Nov 14;5(4):zcad053. doi: 10.1093/narcan/zcad053 (PMC10644876; doi:10.1093/narcan/zcad053)
Supplement: zcad053_Supplemental_Files [file zcad053_supplemental_files.zip › Supplementary Figure_revised.pdf]

Figure S1

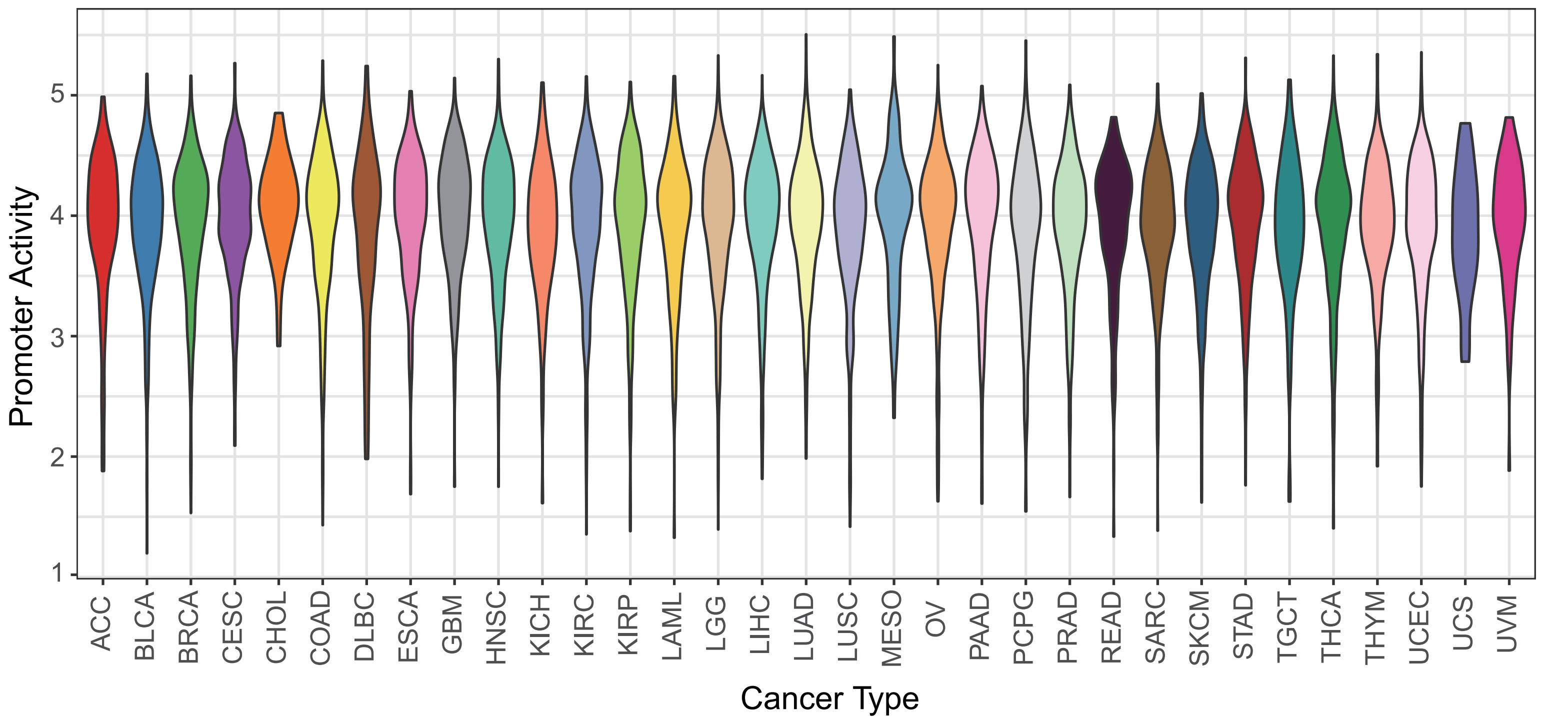

Figure S1. Distribution of promoter activity across cancer types.

Figure S2

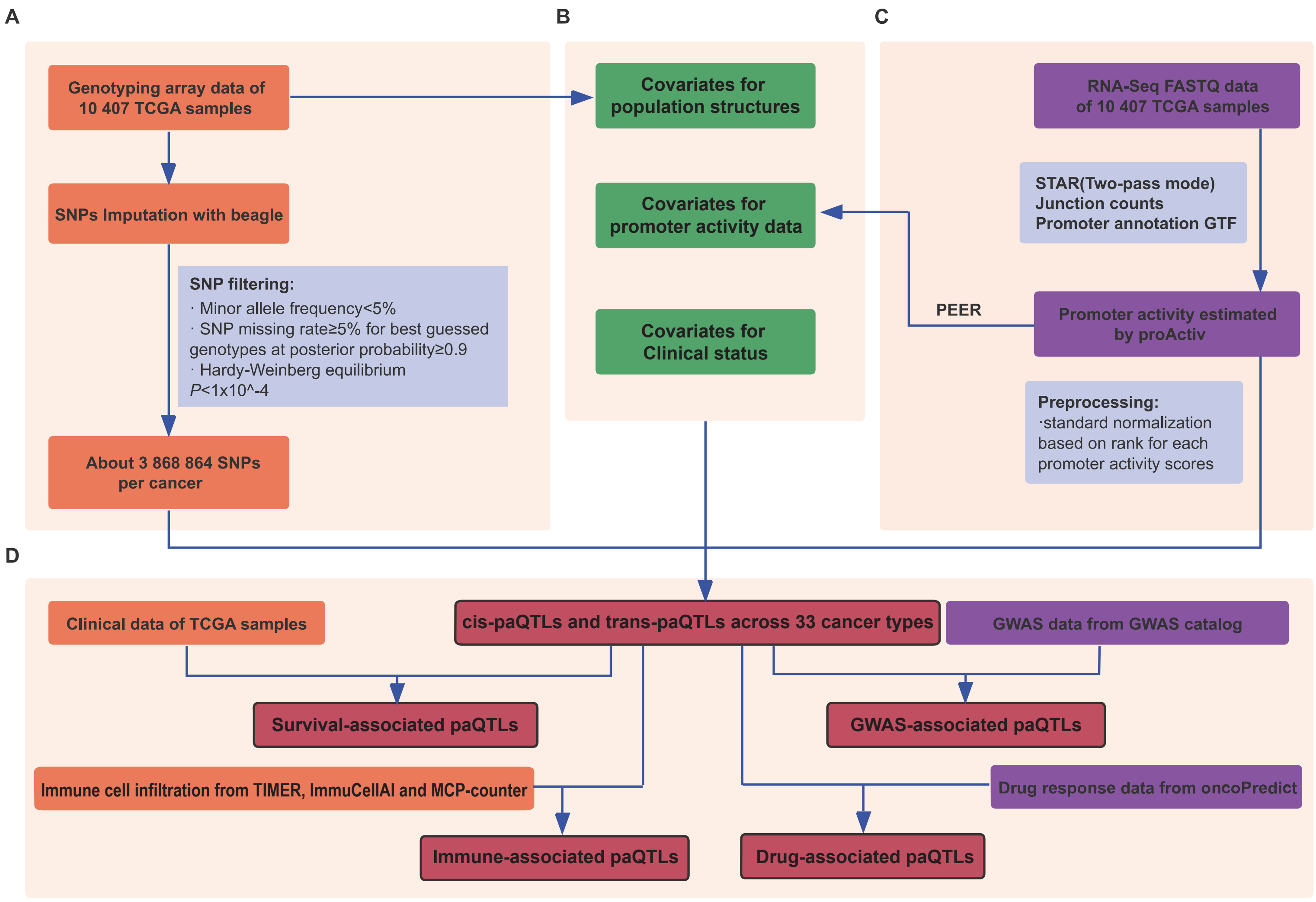

Figure S2. Workflow of Pancan-paQTL in characterization of paQTLs, Survival-paQTLs associations, GWAS-paQTLs associations, Immune-paQTLs associations and Drug-paQTLs ssociations across cancer types.

Figure S3

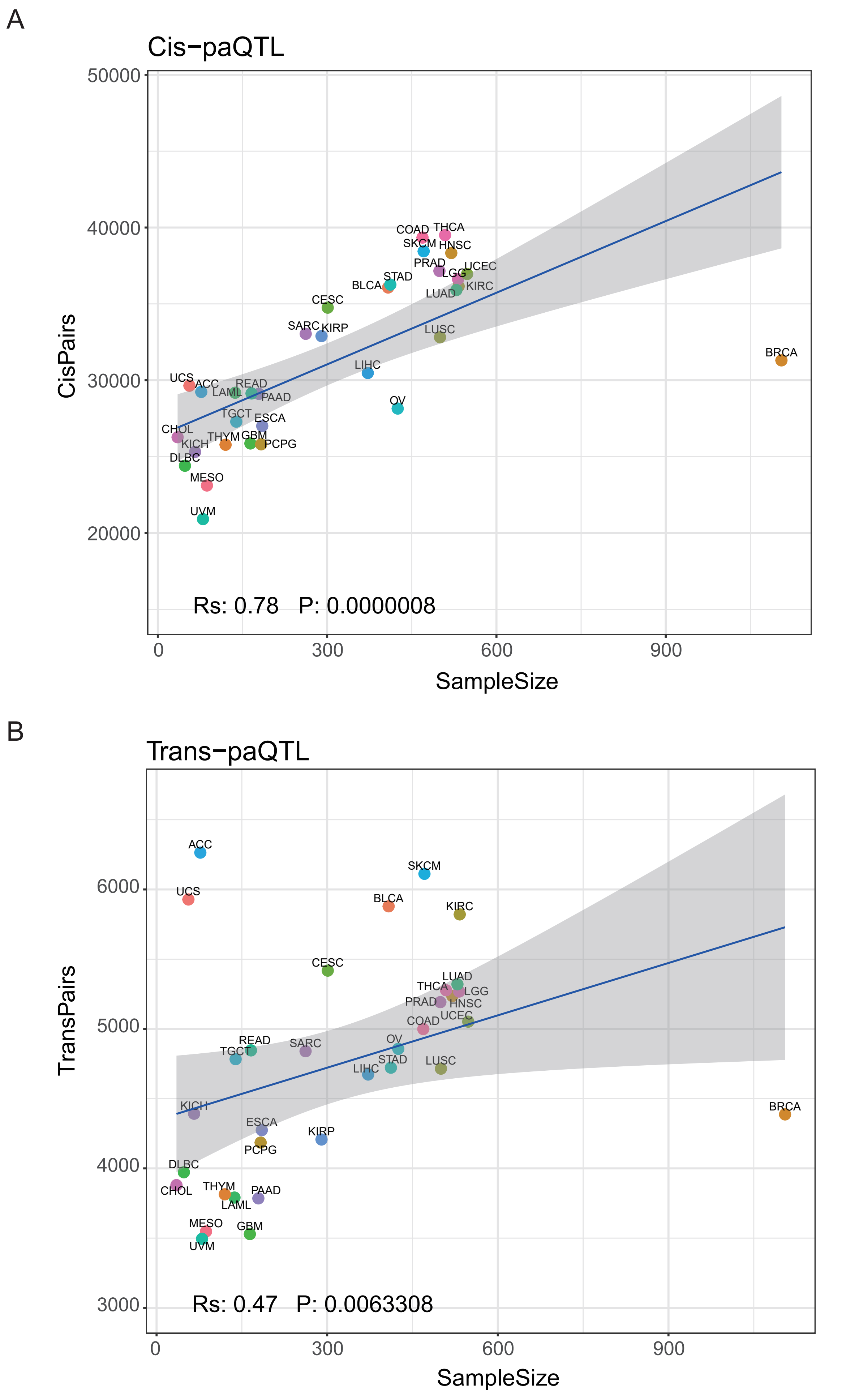

Figure S3. Correlation between sample size and Cis-paQTL pairs (A), Trans-paQTL pairs (B) across cancer types.

Figure S4

A

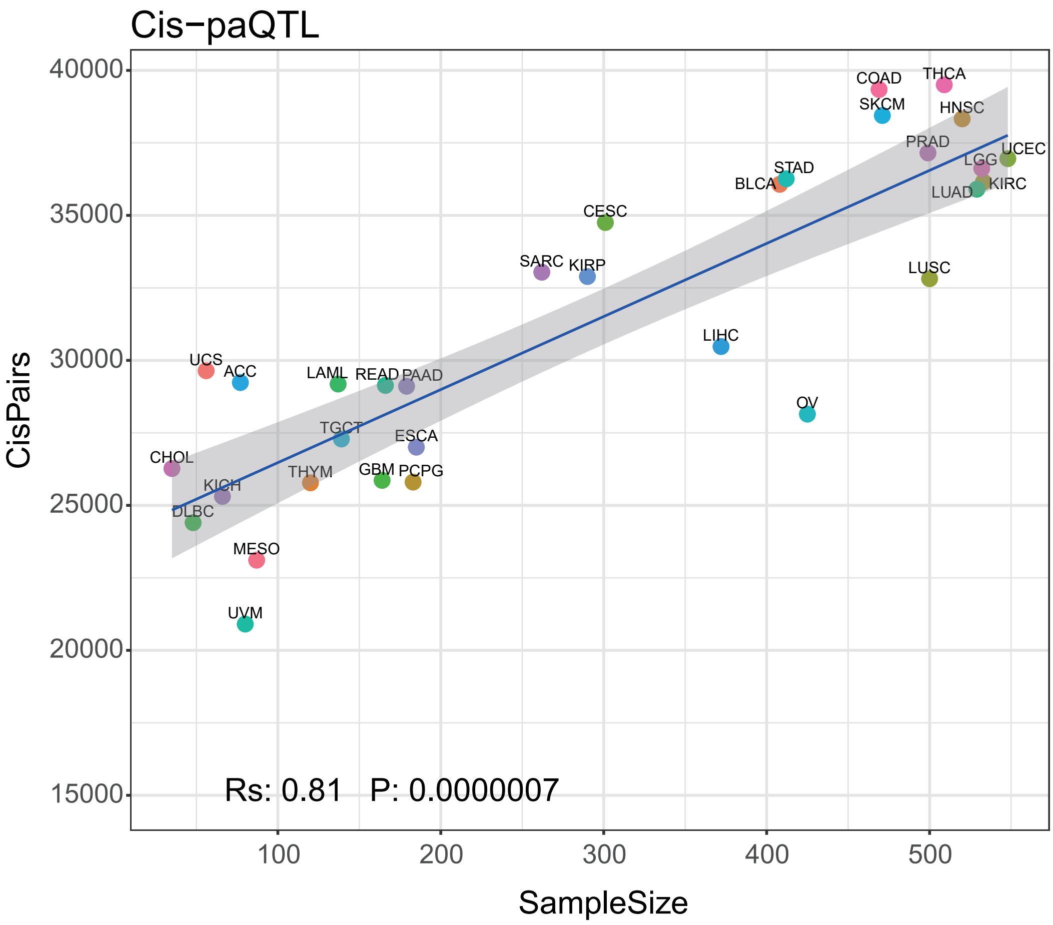

B

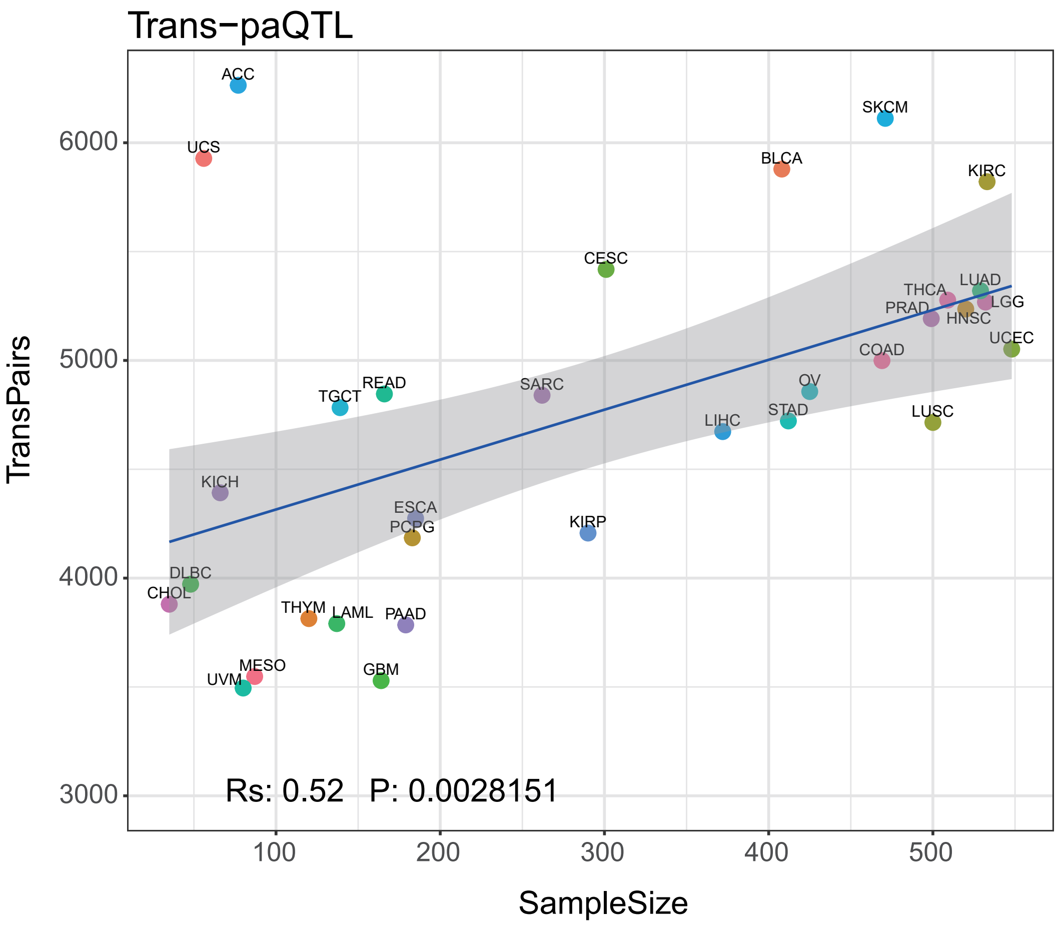

Figure S4. Correlation between sample size and Cis-paQTL pairs (A), Trans-paQTL pairs (B) across cancer types without BRCA.

Figure S5

Pancan-paQTL: Pan-cancer promoter activity Quantitative Trait Loci

A database to evaluate the effects of SNPs on promoter activity

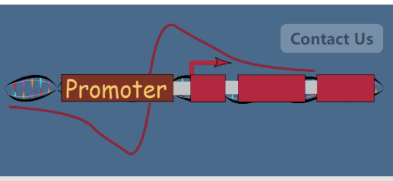

Contact Us

HOME | Cis-paQTL | Trans-paQTL | Survival-paQTL | GWAS-paQTL | Drug-paQTL | Immune-paQTL | Download | Help

◆ Search results for Cis-paQTL

NO.	Cancer	Type	SNP	SNP position	Alleles	Promoter ID	Promoter position	Ensembl ID	Gene Symbol	beta	t-stat	Pvalue	FDR	Figure
1	TCGA-ACC	cis	rs9298753	chr9:16267196	A/T	prmtr_49950	chr9:15874541	ENSG00000164989	CCDC171	0.89	4.68	2.1e-05	9.4e-04	
2	TCGA-ACC	cis	rs12003179	chr9:16265151	C/T	prmtr_49950	chr9:15874541	ENSG00000164989	CCDC171	0.81	4.40	5.3e-05	9.4e-04	
3	TCGA-ACC	cis	rs276454	chr9:15585049	A/G	prmtr_49950	chr9:15874541	ENSG00000164989	CCDC171	0.64	4.33	6.8e-05	9.4e-04	
4	TCGA-ACC	cis	rs7867833	chr9:15536857	A/T	prmtr_49950	chr9:15874541	ENSG00000164989	CCDC171	0.58	3.92	2.6e-04	9.7e-04	
5	TCGA-ACC	cis	rs3008748	chr9:15969472	C/T	prmtr_49950	chr9:15874541	ENSG00000164989	CCDC171	-0.51	-3.81	3.7e-04	9.8e-04	
6	TCGA-ACC	cis	rs276447	chr9:15581304	A/G	prmtr_49950	chr9:15874541	ENSG00000164989	CCDC171	0.54	3.79	3.9e-04	9.8e-04	
7	TCGA-ACC	cis	rs2987070	chr9:15942767	A/G	prmtr_49950	chr9:15874541	ENSG00000164989	CCDC171	0.52	3.78	4.0e-04	9.8e-04	
8	TCGA-ACC	cis	rs2987072	chr9:15944450	C/T	prmtr_49950	chr9:15874541	ENSG00000164989	CCDC171	0.53	3.77	4.1e-04	9.8e-04	
9	TCGA-ACC	cis	rs1328265	chr9:15978633	A/G	prmtr_49950	chr9:15874541	ENSG00000164989	CCDC171	-0.54	-3.76	4.3e-04	9.8e-04	
10	TCGA-ACC	cis	rs140382641	chr9:15597151	C/T	prmtr_49950	chr9:15874541	ENSG00000164989	CCDC171	0.60	3.73	4.8e-04	9.8e-04	
11	TCGA-ACC	cis	rs2457263	chr9:15599352	G/T	prmtr_49950	chr9:15874541	ENSG00000164989	CCDC171	0.60	3.73	4.8e-04	9.8e-04	
12	TCGA-ACC	cis	rs432452	chr9:15602070	A/T	prmtr_49950	chr9:15874541	ENSG00000164989	CCDC171	0.60	3.73	4.8e-04	9.8e-04	
13	TCGA-ACC	cis	rs4741514	chr9:15599353	A/G	prmtr_49950	chr9:15874541	ENSG00000164989	CCDC171	0.60	3.73	4.8e-04	9.8e-04	
14	TCGA-ACC	cis	rs3008666	chr9:15961573	A/C	prmtr_49950	chr9:15874541	ENSG00000164989	CCDC171	-0.53	-3.72	4.8e-04	9.8e-04	

◆ Search results for GWAS-paQTL

NO.	Cancer	Type	Traits	tagSNP	LD	SNP	SNP position	Alleles	Promoter ID	Promoter position	Ensembl ID	Gene symbol	beta	t-stat	Pvalue	FDR
1	TCGA-ACC	cis	Acceptance of an invitation to participate in a mental health questionnaire	rs12340969	0.60	rs276454	chr9:15585049	A/G	prmtr_49950	chr9:15874541	ENSG00000164989	CCDC171	0.64	4.33	6.8e-05	9.4e-04
2	TCGA-ACC	cis	Body mass index	rs4740619	0.63	rs276454	chr9:15585049	A/G	prmtr_49950	chr9:15874541	ENSG00000164989	CCDC171	0.64	4.33	6.8e-05	9.4e-04
3	TCGA-ACC	cis	Body mass index	rs9776097	0.53	rs276454	chr9:15585049	A/G	prmtr_49950	chr9:15874541	ENSG00000164989	CCDC171	0.64	4.33	6.8e-05	9.4e-04
4	TCGA-ACC	cis	Acceptance of an invitation to participate in a mental health questionnaire	rs12340969	0.50	rs7867833	chr9:15536857	A/T	prmtr_49950	chr9:15874541	ENSG00000164989	CCDC171	0.58	3.92	2.6e-04	9.7e-04
5	TCGA-ACC	cis	Acceptance of an invitation to participate in a mental health questionnaire	rs12340969	0.51	rs7867900	chr9:15536669	A/G	prmtr_49950	chr9:15874541	ENSG00000164989	CCDC171	0.58	3.92	2.6e-04	9.7e-04
6	TCGA-ACC	cis	Body mass index	rs4740619	0.54	rs7867833	chr9:15536857	A/T	prmtr_49950	chr9:15874541	ENSG00000164989	CCDC171	0.58	3.92	2.6e-04	9.7e-04
7	TCGA-ACC	cis	Body mass index	rs4740619	0.55	rs7867900	chr9:15536669	A/G	prmtr_49950	chr9:15874541	ENSG00000164989	CCDC171	0.58	3.92	2.6e-04	9.7e-04
8	TCGA-ACC	cis	Body fat percentage	rs10756713	0.56	rs3008748	chr9:15969472	C/T	prmtr_49950	chr9:15874541	ENSG00000164989	CCDC171	-0.51	-3.81	3.7e-04	9.8e-04
9	TCGA-ACC	cis	Body mass index	rs1927702	0.94	rs3008748	chr9:15969472	C/T	prmtr_49950	chr9:15874541	ENSG00000164989	CCDC171	-0.51	-3.81	3.7e-04	9.8e-04
10	TCGA-ACC	cis	Predicted visceral adipose tissue	rs10756714	0.56	rs3008748	chr9:15969472	C/T	prmtr_49950	chr9:15874541	ENSG00000164989	CCDC171	-0.51	-3.81	3.7e-04	9.8e-04

◆ Search results for Drug-paQTL

NO.	Cancer	Type	SNP	SNP position	Alleles	Promoter ID	Promoter position	Ensembl ID	Gene Symbol	Drug name	Drug target	Target pathway	RS	Pvalue	FDR	Figure
1	TCGA-ACC	cis	rs12003179	chr9:16265151	C/T	prmtr_49950	chr9:15874541	ENSG00000164989	CCDC171	JAK1			0.39	4.2e-04	4.9e-04	
2	TCGA-ACC	cis	rs1328265	chr9:15978633	A/G	prmtr_49950	chr9:15874541	ENSG00000164989	CCDC171	JAK1			0.39	4.2e-04	4.9e-04	
3	TCGA-ACC	cis	rs140382641	chr9:15597151	C/T	prmtr_49950	chr9:15874541	ENSG00000164989	CCDC171	JAK1			0.39	4.2e-04	4.9e-04	
4	TCGA-ACC	cis	rs2457263	chr9:15599352	G/T	prmtr_49950	chr9:15874541	ENSG00000164989	CCDC171	JAK1			0.39	4.2e-04	4.9e-04	
5	TCGA-ACC	cis	rs276447	chr9:15581304	A/G	prmtr_49950	chr9:15874541	ENSG00000164989	CCDC171	JAK1			0.39	4.2e-04	4.9e-04	
6	TCGA-ACC	cis	rs276454	chr9:15585049	A/G	prmtr_49950	chr9:15874541	ENSG00000164989	CCDC171	JAK1			0.39	4.2e-04	4.9e-04	
7	TCGA-ACC	cis	rs2987070	chr9:15942767	A/G	prmtr_49950	chr9:15874541	ENSG00000164989	CCDC171	JAK1			0.39	4.2e-04	4.9e-04	
8	TCGA-ACC	cis	rs2987072	chr9:15944450	C/T	prmtr_49950	chr9:15874541	ENSG00000164989	CCDC171	JAK1			0.39	4.2e-04	4.9e-04	
9	TCGA-ACC	cis	rs3008666	chr9:15961573	A/C	prmtr_49950	chr9:15874541	ENSG00000164989	CCDC171	JAK1			0.39	4.2e-04	4.9e-04	
10	TCGA-ACC	cis	rs3008748	chr9:15969472	C/T	prmtr_49950	chr9:15874541	ENSG00000164989	CCDC171	JAK1			0.39	4.2e-04	4.9e-04	
11	TCGA-ACC	cis	rs432452	chr9:15602070	A/T	prmtr_49950	chr9:15874541	ENSG00000164989	CCDC171	JAK1			0.39	4.2e-04	4.9e-04	
12	TCGA-ACC	cis	rs4741514	chr9:15599353	A/G	prmtr_49950	chr9:15874541	ENSG00000164989	CCDC171	JAK1			0.39	4.2e-04	4.9e-04	
13	TCGA-ACC	cis	rs7867833	chr9:15536857	A/T	prmtr_49950	chr9:15874541	ENSG00000164989	CCDC171	JAK1			0.39	4.2e-04	4.9e-04	
14	TCGA-ACC	cis	rs9298753	chr9:16267196	A/T	prmtr_49950	chr9:15874541	ENSG00000164989	CCDC171	JAK1			0.39	4.2e-04	4.9e-04	

◆ Search results for Immune-paQTL

NO.	Cancer	Type	SNP	SNP position	Alleles	Promoter ID	Promoter position	Ensembl ID	Gene Symbol	Immune cell	Source	RS	Pvalue	FDR	Figure
1	TCGA-ACC	cis	rs12003179	chr9:16265151	C/T	prmtr_49950	chr9:15874541	ENSG00000164989	CCDC171	Macrophage	ImmuCellAI	-0.40	2.2e-04	4.9e-04	
2	TCGA-ACC	cis	rs12005048	chr9:16265065	A/G	prmtr_49950	chr9:15874541	ENSG00000164989	CCDC171	Macrophage	ImmuCellAI	-0.40	2.2e-04	4.9e-04	
3	TCGA-ACC	cis	rs1328265	chr9:15978633	A/G	prmtr_49950	chr9:15874541	ENSG00000164989	CCDC171	Macrophage	ImmuCellAI	-0.40	2.2e-04	4.9e-04	
4	TCGA-ACC	cis	rs140382641	chr9:15597151	C/T	prmtr_49950	chr9:15874541	ENSG00000164989	CCDC171	Macrophage	ImmuCellAI	-0.40	2.2e-04	4.9e-04	
5	TCGA-ACC	cis	rs1539172	chr9:15784633	A/G	prmtr_49950	chr9:15874541	ENSG00000164989	CCDC171	Macrophage	ImmuCellAI	-0.40	2.2e-04	4.9e-04	
6	TCGA-ACC	cis	rs2457263	chr9:15599352	G/T	prmtr_49950	chr9:15874541	ENSG00000164989	CCDC171	Macrophage	ImmuCellAI	-0.40	2.2e-04	4.9e-04	
7	TCGA-ACC	cis	rs2457265	chr9:15599160	A/G	prmtr_49950	chr9:15874541	ENSG00000164989	CCDC171	Macrophage	ImmuCellAI	-0.40	2.2e-04	4.9e-04	
8	TCGA-ACC	cis	rs276447	chr9:15581304	A/G	prmtr_49950	chr9:15874541	ENSG00000164989	CCDC171	Macrophage	ImmuCellAI	-0.40	2.2e-04	4.9e-04	
9	TCGA-ACC	cis	rs276454	chr9:15585049	A/G	prmtr_49950	chr9:15874541	ENSG00000164989	CCDC171	Macrophage	ImmuCellAI	-0.40	2.2e-04	4.9e-04	
10	TCGA-ACC	cis	rs2987070	chr9:15942767	A/G	prmtr_49950	chr9:15874541	ENSG00000164989	CCDC171	Macrophage	ImmuCellAI	-0.40	2.2e-04	4.9e-04	
11	TCGA-ACC	cis	rs2987072	chr9:15944450	C/T	prmtr_49950	chr9:15874541	ENSG00000164989	CCDC171	Macrophage	ImmuCellAI	-0.40	2.2e-04	4.9e-04	
12	TCGA-ACC	cis	rs3008666	chr9:15961573	A/C	prmtr_49950	chr9:15874541	ENSG00000164989	CCDC171	Macrophage	ImmuCellAI	-0.40	2.2e-04	4.9e-04	

Figure S5. Examples of output using search function by input Promoter ID in ACC.

Figure S6

Pancan-paQTL: Pan-cancer promoter activity Quantitative Trait Loci

A database to evaluate the effects of SNPs on promoter activity

Contact Us

HOME | Cis-paQTL | Trans-paQTL | Survival-paQTL | GWAS-paQTL | Drug-paQTL | Immune-paQTL | Download | Help

◆ Search results for Cis-paQTL

Note: 1 results in current criterion.

1

NO.	Cancer	Type	SNP	SNP position	Alleles	Promoter ID	Promoter position	Ensembl ID	Gene Symbol	beta	t-stat	Pvalue	FDR	Figure
1	TCGA-ACC	cis	rs3008666	chr9:15961573	A/C	prmtr_49950	chr9:15874541	ENSG00000164989	CCDC171	-0.53	-3.72	4.8e-04	9.8e-04	

1

◆ Search results for GWAS-paQTL

Note: 1 results in current criterion.

1

NO.	Cancer	Type	Traits	tagSNP	LD	SNP	SNP position	Alleles	Promoter ID	Promoter position	Ensembl ID	Gene symbol	beta	t-stat	Pvalue	FDR
1	TCGA-ACC	cis	Body mass index	rs1927702	0.88	rs3008666	chr9:15961573	A/C	prmtr_49950	chr9:15874541	ENSG00000164989	CCDC171	-0.53	-3.72	4.8e-04	9.8e-04

1

◆ Search results for Drug-paQTL

Note: 1 results in current criterion.

1

NO.	Cancer	Type	SNP	SNP position	Alleles	Promoter ID	Promoter position	Ensembl ID	Gene Symbol	Drug name	Drug target	Target pathway	RS	Pvalue	FDR	Figure
1	TCGA-ACC	cis	rs3008666	chr9:15961573	A/C	prmtr_49950	chr9:15874541	ENSG00000164989	CCDC171	JAK1			0.39	4.2e-04	4.9e-04	

1

◆ Search results for Immune-paQTL

Note: 1 results in current criterion.

1

NO.	Cancer	Type	SNP	SNP position	Alleles	Promoter ID	Promoter position	Ensembl ID	Gene Symbol	Immune cell	Source	RS	Pvalue	FDR	Figure
1	TCGA-ACC	cis	rs3008666	chr9:15961573	A/C	prmtr_49950	chr9:15874541	ENSG00000164989	CCDC171	Macrophage	ImmuCellAI	-0.40	2.2e-04	4.9e-04	

1

Figure S6. Examples of output using search function by input SNP ID in ACC.
